# Application of Fuzzy Logic for Selection of Actor Nodes in WSANs —Implementation of Two Fuzzy-Based Systems and a Testbed †

**DOI:** 10.3390/s19245573

**Published:** 2019-12-17

**Authors:** Donald Elmazi, Miralda Cuka, Makoto Ikeda, Keita Matsuo, Leonard Barolli

**Affiliations:** 1 Department of Information and Communication Engineering, Fukuoka Institute of Technology (FIT), Fukuoka 811-0295, Japan; makoto.ikd@acm.org (M.I.); kt-matsuo@fit.ac.jp (K.M.); barolli@fit.ac.jp (L.B.); 2 Graduate School of Engineering, Fukuoka Institute of Technology (FIT), Fukuoka 811-0295, Japan; mcuka91@gmail.com

**Keywords:** WSANs, intelligent systems, intelligent algorithms, fuzzy logic, transmission range, load balancing, testbed

## Abstract

The development of sensor networks and the importance of smart devices in the physical world has brought attention to Wireless Sensor and Actor Networks (WSANs). They consist of a large number of static sensors and also a few other smart devices, such as different types of robots. Sensor nodes have responsibility for sensing and sending information towards an actor node any time there is an event that needs immediate intervention such as natural disasters or malicious attacks in the network. The actor node is responsible for processing and taking prompt action accordingly. But in order to select an appropriate actor to do one task, we need to consider different parameters, which make the problem NP-hard. For this reason, we consider Fuzzy Logic and propose two Fuzzy Based Simulation Systems (FBSS). FBSS1 has three input parameters such as Number of Sensors per Actor (NSA), Remaining Energy (RE) and Distance to Event (DE). On the other hand, FBSS2 has one new parameter—Transmission Range (TR)—and for this reason it is more complex. We will explain in detail the differences between these two systems. We also implement a testbed and compare simulation results with experimental results.

## 1. Introduction

Wireless Sensor Networks (WSNs) can be defined as a group of tiny wireless self-programmable devices, easily adaptable, which randomly connect to each other without needing any centralized authority, therefore dynamically sending the data to the intended recipient about the monitored phenomenon [1,2,3].

The development in sensor networks and the importance of smart devices in the physical world has brought attention to Wireless Sensor and Actor Networks (WSANs). They consist of a large number of static sensors and also a few other smart devices, such as different types of robots. WSANs have greatly enhanced the existing wireless sensor networks (WSNs) by its heterogeneous node structure. WSANs monitor their surroundings, process the gathered data, make a proper decision based on these data and complete different tasks when needed.

They can be applied in emergency scenarios such as fire situations, natural disasters and so forth. For, example when a fire happens, sensors detect data such as the position and intensity of the fire and forward it to actors who coordinate with each-other to decide what will extinguish it in time. Actors are different from each other, and some are more suitable for the specific disaster situation. To better illustrate this, let us take into consideration a fire happening on the higher floors of a building and in order to deal with this as soon as possible, a flying type robot will be selected [4,5].

In conventional WSNs, there are two main devices—sensors and sinks. Any changes in the environment will be sensed by the sensors immediately. At a certain time, a task which needs an action happens within the sensing range of the sensors, and they will forward this data to the sink. The sink is a collecting and processing device. Therefore the sink placement has a great impact on the performance of the network. Different from WSNs, where sensors forward all the data to the sink by multi-hop communication, in WSANs, two new interaction types are introduced. After the event is sensed, the actor in the proximity of this sensor will get, collect and then process these data before taking action upon the event. This is the scenario of sensor–actor interaction. In the above situation, before the actor takes action, it will first check if any other actor is better suited for taking over the task to ensure better resource management in the network. This is called actor–actor interaction, which is not only important during task fulfillment, but also during a network’s self-healing situations, that is, connectivity establishment [6,7], reliable services [8], Quality of Service (QoS) [9,10,11] and so on.

For selecting a proper actor to carry out a job we consider different parameters such as transmission range, load balancing, remaining energy and distance to event.

Since the actor and sensors are distributed in an arbitrary way, the main issues include coverage and communication, because of the different obstacles encountered. In general, this reflects how good the coverage is of the nodes in an area. So, to have better and clearer communication, we need to have an average transmission range in order not to affect the energy of the nodes and also to have better quality for communication.

The role of Load Balancing in WSANs is to provide a reliable service. Load balancing identifies the optimal load on nodes of the network to increase the network efficiency.

To have a better network efficiency and also to increase the network lifetime we have considered the parameter of the Remaining Energy (RE). As the devices are active and deployed in many scenarios, they perform tasks and exchange data between them. Due to this and due to their characteristics, some devices may have a lot of energy remaining in them and some others may have less. So, in order to increase the lifetime of the network, our system uses the actor nodes that are closer to the event.

The number of the actors in WSANs is smaller than the number of sensors. Thus, when an actor is called to action to perform a task, their distance to the event differs for every actor. Depending on the distance levels, our system takes decisions on the availability of each actor node.

However, having three or more parameters makes the problem NP-hard. There are different approaches and intelligent systems that can be used to deal with NP-hard problems. In our previous work, we considered Genetic Algorithms (GAs), Neural Networks (NNs), Particle Swarm Optimization (PSO), Hill Climbing (HC), Simulated Annealing (SA) and Tabu Search [12,13,14,15]. However, different intelligent algorithms have different efficiency for different problems. From our experience, the GAs provide good results for optimization and allocation problems. NNs provide good results for rule learning and recognition problems. While Fuzzy logic (FL) can be used for decision making and control problems. For this reason, we consider FL in this paper. Also, by using linguistic variables and a fuzzy rule base, it is easy to express previous knowledge and heuristics in the decision making process. We have proposed and implemented two decision-making systems, which consider uncertainty and vagueness of the data that cannot be computed by mathematical models. The core of Fuzzy Logic is Fuzzy Logic Controller (FLC), which is composed by four units such as the fuzzifier, inference engine, rule base and the defuzzifier. The crisp input value is fuzzified by the fuzzifier into a fuzzy value. By using this value and the rule base, the inference engine infers a fuzzy value, which is defuzzified by the defuzzifier and calculates a crisp value appropriate for control.

In our work, we consider three input linguistic parameters for FBSS1 and four input linguistic parameters for FBSS2. We performed simulations in order to evaluate the performance of the proposed systems. We also implemented a testbed and compared simulation results with experimental results. The comparison evaluation shows that experimental results are close to the simulation results, but there are some oscillations.

Different to our other published works [16,17,18], in this work we have added Number of Sensors per Actor (NSA) and Transmission Range (TR) to make the comparison between two fuzzy-based systems and also to evaluate the simulation results of one of the systems with the experimental results.

The organization of the paper is as follows. The basics of WSANs including challenges and architecture are described in Section 2. The two systems and their implementation are described in Section 3. The experimental set up is explained in Section 4. Simulation and Experimental results are reported in Section 5. Finally, the paper is concluded in Section 6.

## 2. WSANs

### 2.1. WSANs Challenges

Some of the issues in WSANs related to the actors’ features are listed below.

- *Actor and Sensor Placement*: To avoid isolated islands being created and coverage only of secluded areas, a uniform deployment of sensors and actors is a major concern as the performance of the networks depends on it. By having a better spatially distributed network, we make better use of the collected data [19,20].
- *Real-Time*: WSANs are a distributed control system that requires a real-time reaction for the information gathered by the sensors. In case of an emergency situation the real-time response is crucial to guarantee immediate action [21].
- *Coordination*: Unlike WSNs, where the sink performs the functions of the gathered data, a new phenomena occurs in WSANs called sensor–actor and actor–actor coordination. This phenomenon provides the transmission of the event features from sensors to actors [22].
- *Energy Efficiency*: Each task, based on the difficulty, consumes power and drains the battery of the sensors. In order to increase the lifetime of the network, we should consider the energy of the sensors since they are power-constrained devices.
- *Scalability*: In a rapidly changing environment, it is a very big challenge for the network to meet scalability needs. A WSAN should have the capability to handle the growth of affecting the existing network [20,23].

### 2.2. WSAN Architecture

**Figure 1:**
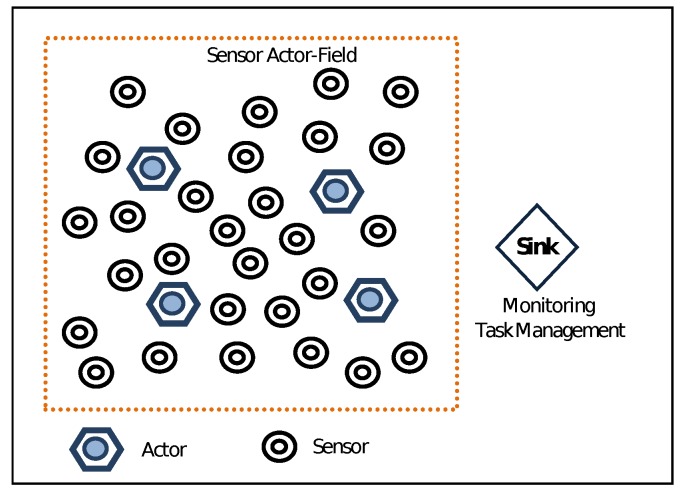
Wireless Sensor Actor Networks (WSANs).

**Figure 2:**
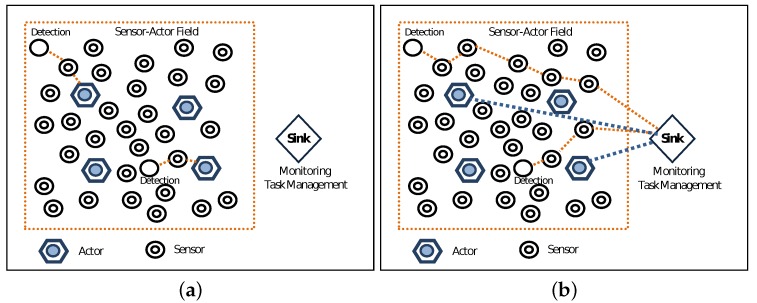
Architectures of WSANs. (**a**) Fully-Automated. (**b**) Semi-Automated.

A WSAN is shown in Figure 1. In WSANs, the information of the physical environment is gathered by the sensors while the appropriate action upon the environment and the decision making is performed by the actors. The actor, besides being an acting device, is also an entity that performs network functionalities such as data receiving, processing and transmitting. After the sensors gather the data from the event, the action upon it depends on whether we have Semi-Automated or Fully-Automated architecture (see Figure 2). In the first case, the sensors forward the data to the sink. The sink is a resource-rich processing device, which collects the data and coordinates the devices.

In the Fully-Automated architecture, the sensors forward the data directly to the nearest actor. Afterward, the actors communicate with each other and coordinate to choose which of them will perform the task. This type of architecture is more complex as it needs sophisticated algorithms. Compared to the Semi-Automated architecture, it has some advantages such as long network lifetime, higher reliability, low energy consumption [4], low latency, and so on.

## 3. Proposed Fuzzy-Based Systems

After an event detection, the act upon this event depends on the present architecture type. These types of networks are characterized by the heterogeneity of the actors. This means that, based on the event type, we select the most suitable actor to perform this task.

### 3.1. System Parameters

Considering the challenges of WSANs, we have decided to use the following parameters for implementing our proposed system.

**Transmission Range (TR):** A well connected network is when all the devices are within the transmission range of each other. This parameter affects the network topology. A high range will achieve connection between nodes at the expense of high consumption of energy. A shot range will use less energy to forward the data, but more hops are required to reach the destination. So, in our system we have considered an average transmission range.

**Distance to Event (DE):** Our system considers the availability of the actors when needed for action near an event. The distance of the actor to the event varies for different scenarios.

**Remaining Energy (RE):** These devices are power-constrained and one of the main issues is power consumption. Each task consumes different amounts of energy depending on its difficulty. Therefore, is very important to utilize the remaining energy of each node efficiently.

**Number of Sensors per Actor (NSA):** It is important that all actors are within the sensing range of the sensors. In some situations the actors are secluded with a low number of sensors which decreases the chances of the event being detected. On the other hand, having too many sensors per actor isolates the network to a specific area. So it is better to have a balanced number of sensors within the range of an actor.

**Actor Selection Decision (ASD):** Based on the input parameters, our system makes a decision on which actor is to be selected to perform the task.

Parameters vary in different time steps depending on whether actors have or have not used their resources. With our simulation system, we aim to solve problems that are difficult for human reasoning. It should be noted that the problems with more than two unrelated parameter combinations are NP-Hard.

### 3.2. Implementation of Fuzzy Logic

We have implemented fuzzy logic in the area of actor selection for WSAN. Fuzzy logic uses an approximation approach for decision making [24,25,26,27,28,29,30,31,32,33,34,35,36,37,38,39,40].

The systems are implemented directly in the actors because they have high computational resources. Sensors are devices which detect, gather and distribute the data. In the case of Fully Automated Architecture, as mentioned above, the actors have the ability to communicate with each other for making the decision. On the other hand in the Semi-Automated Architecture, a centralized administration tool (Sink), analyzes and makes the decision.

**Figure 3:**
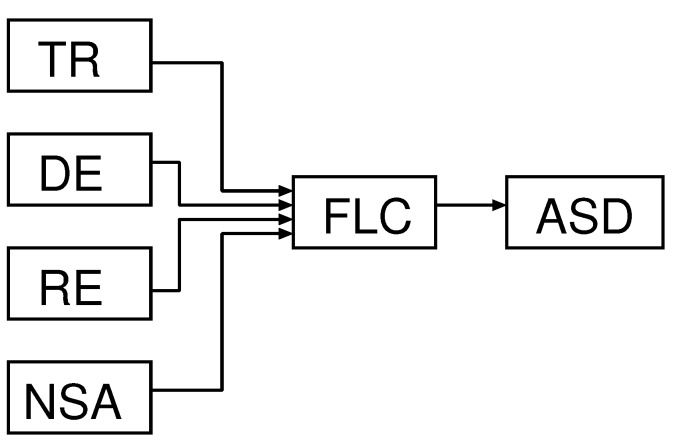
Proposed System.

**Figure 4:**
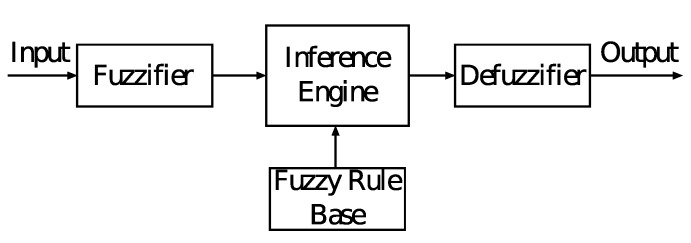
Fuzzy Logic Controller.

Our proposed system is shown in Figure 3. The crisp input parameters (TR, NSA, RE, DE) are inserted on the fuzzy logic controller, which is shown in Figure 4.

We have implemented two systems based on Fuzzy theory, one with 3 input parameters and the other one with 4 input parameters. We have assigned 3 linguistic values for each parameter. This is because there are different scenarios and applications that require different parameters.

**Figure 5:**
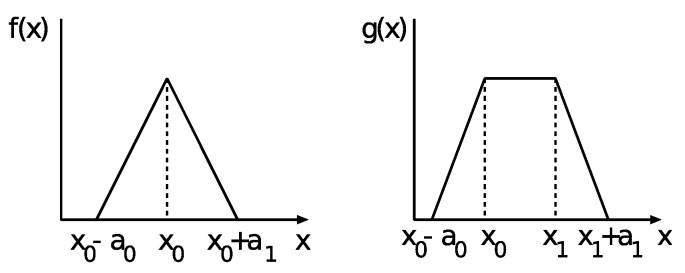
Membership functions types.

The membership functions measure the linguistic terms’ and maps’ non-fuzzy input to fuzzy and vice versa. There are different types of membership functions which are used for different applications. The types of membership functions we have used are shown in Figure 5. As can be seen from the figures, one value can be defined by more than one set and can have two different values at the same time.

### 3.3. Description of FBSS1 and FBSS2

We use three input parameters for FBSS1:

- Distance to Event (DE);
- Remaining Energy (RE);
- Number of Sensors per Actor (NSA).

For FBSS2, we add a new parameter together with the FBSS1 parameters:

- Transmission Range (TR).

**Figure 6:**
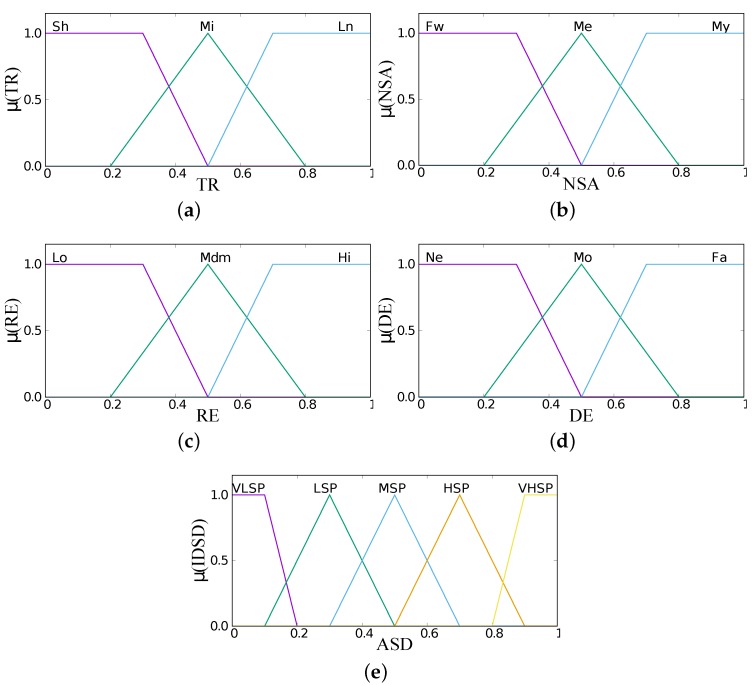
Fuzzy membership functions. (**a**) Transmission Range. (**b**) Number of Sensors per Actor. (**c**) Remaining Energy. (**d**) Distance to Event. (**e**) Actor Selection Decision.

The term sets for each input linguistic parameter are defined respectively as shown in Table 1. Input and output membership functions are shown in Figure 6. After the input parameters are fuzzified, the fuzzy controller will decide which actor will be selected based on the membership functions and the fuzzy rule matrix of combinations number and input parameters. The table for FRB1 is shown in Table 2, and we have a total of twenty-seven rules. For the FRB2 we have eighty-one rules, as shown in Table 3, for four input parameters and three term sets each. The conditional statements of “IF–THEN” are used for the control rules.

## 4. Testbed Description

**Figure 7:**
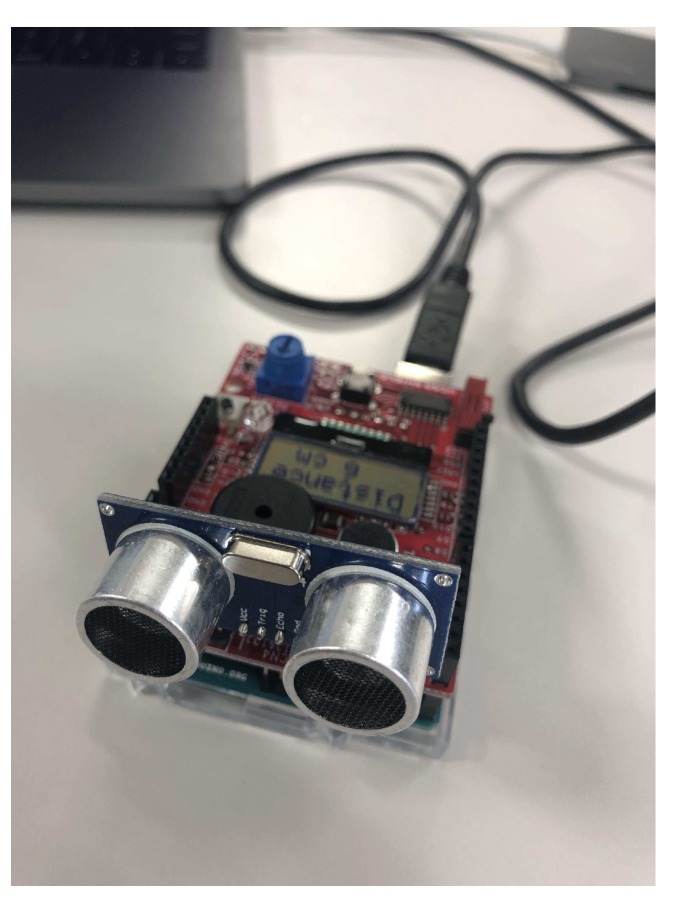
A distance measuring sensor.

**Figure 8:**
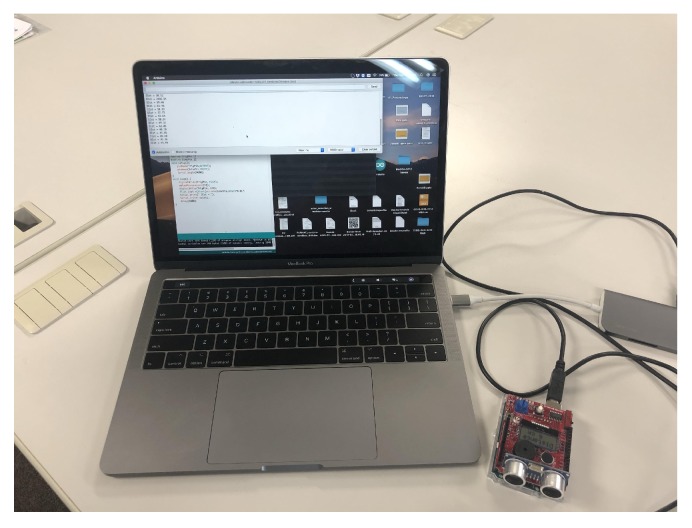
Workstation.

An experiment was conducted to evaluate this simulation system. We selected an indoor environment and placed the sensors and actors randomly. Three different types of sensors (see Figure 7) were mounted on Arduino Uno (see Figure 8), which acted as actors. The event was also randomly deployed. The actors did different measures for different event positions. In order to move the actors freely, some of them were connected to a Raspberry Pi 3 B+, but in this scenario the battery consumption was higher than when we used a laptop as the processing unit.

- The transmission range of the actor.
- A power bank or laptop battery to check the levels of remained energy after each measurement.
- Distance sensor to measure the distance from actor to the event.

The experimental parameters are shown in the Table 4.

## 5. Simulation and Experimental Results

In this section, we present the simulation results. The simulations are carried out in a Linux Ubuntu OS computer with these specifications—RAM (8 GB), CPU i5 (3.2 GHz × 4) and SSD (650 GB). For simulation, we used our implemented FuzzyC system. The FuzzyC is a simulation system written in C language and equipped with Fuzzy library.

### 5.1. Simulation Results

**Figure 9:**
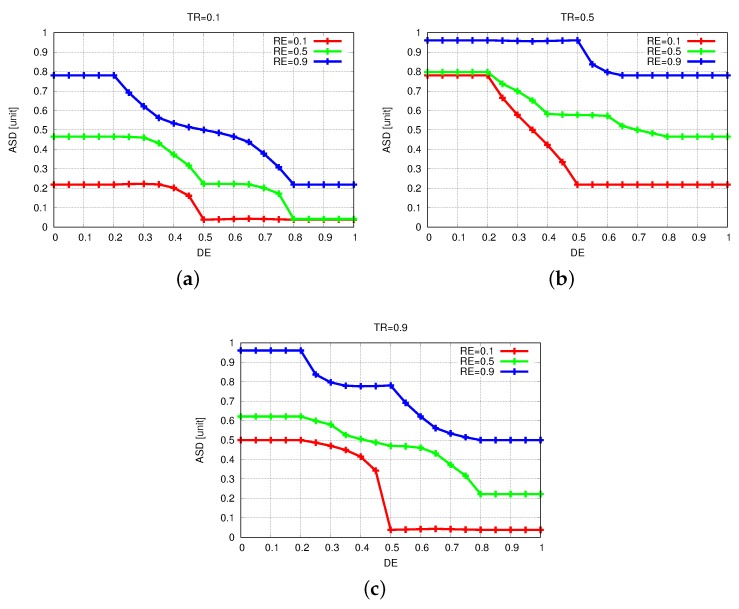
Results for different values of Transmission Range (TR). (**a**) TR = 0.1. (**b**) TR = 0.5. (**c**) TR = 0.9.

We have presented the simulation results of our system in Figure 9. In the networks where nodes are mobile, energy consumption is a main issue. In Figure 9a, TR is 0.1, DE is 0.2 and for RE from 0.1 to 0.5, ASD increases by 27% and when RE goes from 0.5 to 0.9, the ASD is increased by 31%. This means that the lifetime of the network will be longer if actors have a high amount of residual energy.

To see the effect of TR on actor selection we compare Figure 9c with Figure 9b, for RE = 0.1 and DE = 0.2. As expected, the ASD has decreased 29%, because TR is increased from 0.5 to 0.9. In Figure 9c, we observe the effect of DE. For RE = 0.1 and DE from 0.1 to 0.9, ASD has dropped by 49.5%.

**Figure 10:**
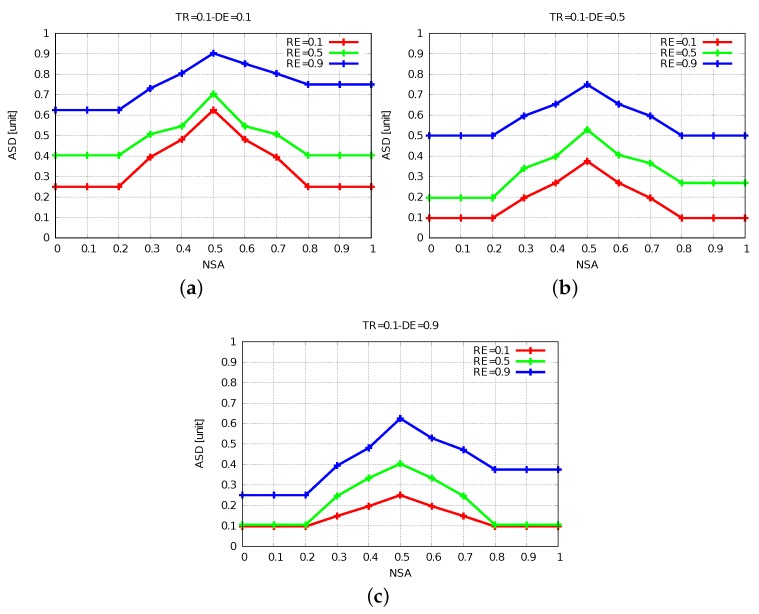
Results for TR = 0.1. (**a**) DE = 0.1. (**b**) DE = 0.5. (**c**) DE = 0.9.

**Figure 11:**
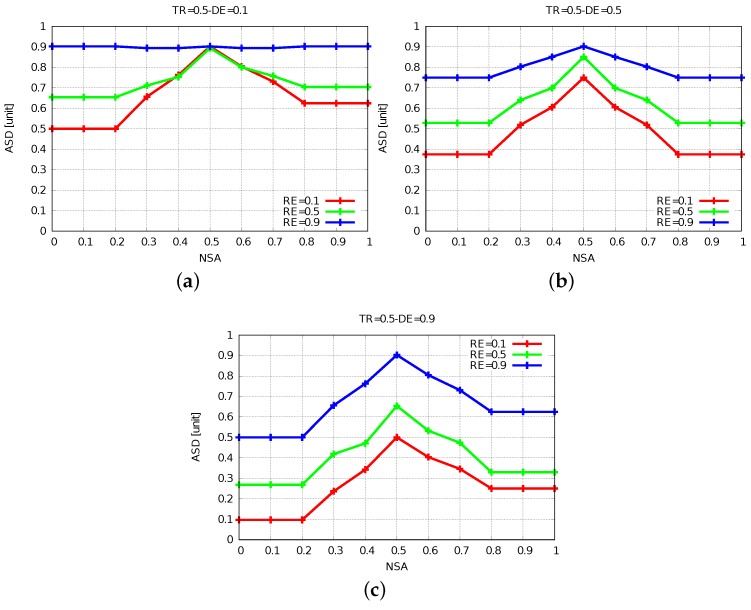
Results for TR = 0.5. (**a**) DE = 0.1. (**b**) DE = 0.5. (**c**) DE = 0.9.

**Figure 12:**
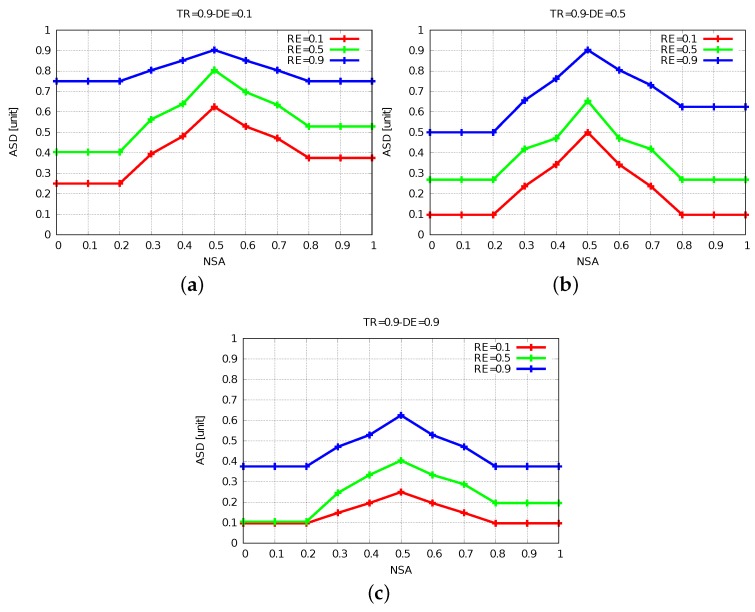
Results for TR = 0.9. (**a**) DE = 0.1. (**b**) DE = 0.5. (**c**) DE = 0.9

The simulation results of FBSS2 are shown in Figure 10, Figure 11 and Figure 12. Having these simulation results, we can say that while DE raises, the ASD declines, because for further distances of events actors have to move far and at the same time they spend a lot more energy. The increase of DE decreases ASD and we show this by comparing Figure 11a with Figure 11b,c. When NSA = 0.5, RE = 0.1 and TR = 0.5 for DE from 0.1 to 0.5 and from 0.1 to 0.9, ASD has decreased by 15% and 40%, respectively.

In WSANs, many clusters of actors and sensors are created, so it is essential to have a balanced distribution of sensors per actor. As shown in Figure 10a, we see the relationship between ASD and NSA, when TR = 0.1 and DE = 0.1. For three selected values of NSA, 0.2, 0.5 and 0.8, we see that NSA is increased by 27% from 0.2 to 0.5 unit and decreased by 15% from 0.5 to 0.8 unit.

Comparing Figure 11b with Figure 10b and Figure 12b with Figure 10b, by increasing TR from 0.1 to 0.5 and 0.9, the ASD is increased by 37% and decreased by 25%, respectively.

Hence, it is important to utilize the power of each actor with efficiency in order to have a longer network lifetime. If the TR is kept low, even when the energy will be consumed a little, the packets might not be able to reach the destination. On the other hand, if the TR is kept high, the energy will be consumed faster, but the coverage will be higher. So, in order to have an optimized network lifetime, an optimal value of TR needs to be chosen.

### 5.2. Experimental Results

**Figure 13:**
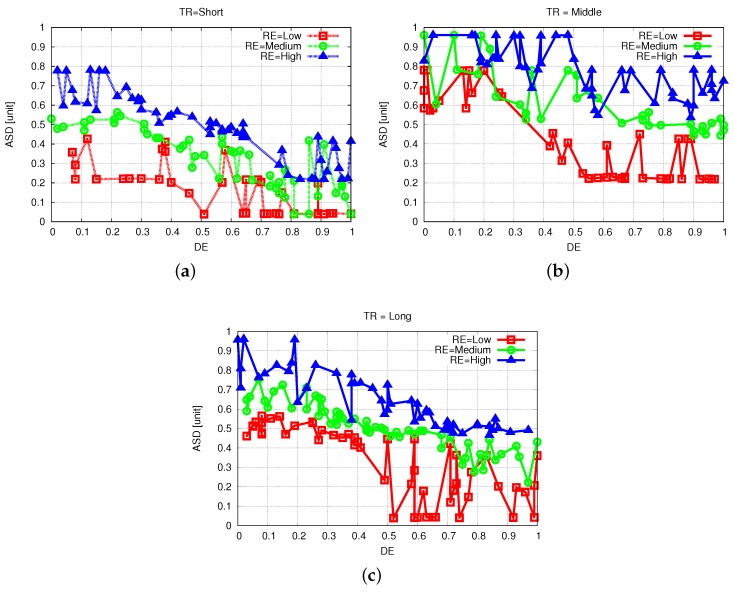
Experimental results for different linguistic variables of TR. (**a**) TR = Short. (**b**) TR = Middle. (**c**) TR = Long.

In this section, we describe the work towards solving the actors selection problem by setting up a testbed for evaluating the results of our system in a real life scenario. We present the experimental results in Figure 13. The three graphs belong to one of each linguistic variable of TR: Short, Middle and Long. The RE is represented with different color lines with blue being the High value, Medium is the green one and red is Low.

By analyzing the experimental results and simulation results, we observed that the curves follow a similar trend with some deviations. Experimental results show that the ASD is increased for higher RE, shorter DE and an average value of TR. The same phenomenon is also observed in the simulation results.

**Figure 14:**
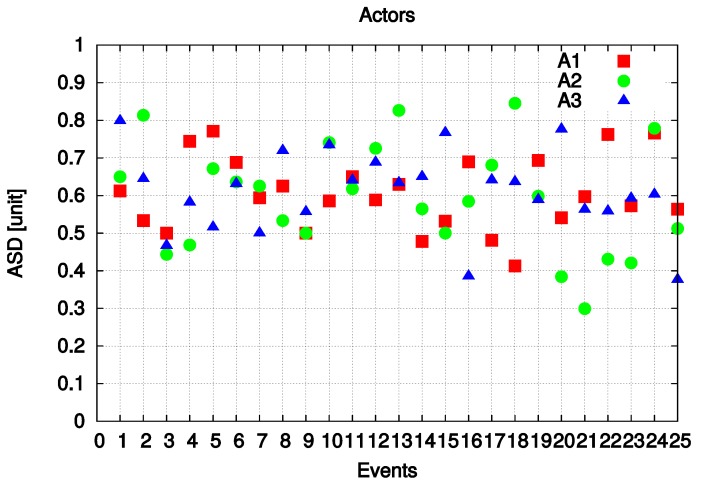
Experimental results for different Events.

Each actor (A1, A2 and A3) parameters were measured for a total of 25 unrelated events (see Figure 14). In different moments, actors have different parameter values conditioned by the always changing topology. Comparing the values of each parameter for the three actors, only one is to be chosen for performing the task. So, we see that in event 3, A1 is selected to do the task. At event 15, A3 is selected and in event 24, A2 is suitable for carrying out the task.

Because there are three and four parameters for making decisions, the ASD values are different, so only one actor is selected.

## 6. Conclusions and Future Work

The conclusions for our proposed systems and testbed are given below. With our systems, we expected actors to complete the task without wasting resources. We observed the effect of these parameters—TR, NSA, RE and DE—on ASD. The further away the actor, the less they are favored to be selected for the task. So an increase in DE results in a decrease in ASD. A higher level of residual energy keeps the actor in the network longer. As for the TR parameter to preserve energy and to limit the number of hops, we saw that an average value is more preferable as it increases ASD. Comparing FBSS1 with FBSS2, the FBSS2 is more complex than FBSS1, but it makes a better selection of actor nodes. A drawback of implementing a testbed is that they are confined to the environment, where simulation results offer a broad range of scenarios. By analyzing the experimental results and simulation results, we observed that the curves follow a similar trend with some deviations.

For future work, we will implement other systems and propose new parameters for actor selection and will conduct extensive experiments and simulations to evaluate the systems.

## Figures and Tables

**Table 1:** Linguistic variables of input and output parameters.

Parameters	Term Sets
Transmission Range (TR)	Short (Sh), Middle (Mi), Long (Ln)
Distance to Event (DE)	Near (Ne), Moderate (Mo), Far (Fa)
Remaining Energy (RE)	Low (Lo), Medium (Mdm), High (Hi)
Number of Sensors per Actor (NSA)	Few (Fw), Medium (Me), Many (My)
Actor Selection Decision (ASD)	Very Low Selection Possibility (VLSP), Low Selection Possibility (LSP), Middle Selection Possibility (MSP), High Selection Possibility (HSP), Very High Selection Possibility (VHSP)

**Table 2:** FRB1.

No.	NSA	RE	DE	ASD
1	Fw	Lo	Ne	LSP
2	Fw	Lo	Mo	VLSP
3	Fw	Lo	Fa	VLSP
4	Fw	Mdm	Ne	MSP
5	Fw	Mdm	Mo	VLSP
6	Fw	Mdm	Fa	VLSP
7	Fw	Hi	Ne	VHSP
8	Fw	Hi	Mo	LSP
9	Fw	Hi	Fa	LSP
10	Me	Lo	Ne	MSP
11	Me	Lo	Mo	VLSP
12	Me	Lo	Fa	VLSP
13	Me	Mdm	Ne	VHSP
14	Me	Mdm	Mo	LSP
15	Me	Mdm	Fa	VLSP
16	Me	Hi	Ne	VHSP
17	Me	Hi	Mo	MSP
18	Me	Hi	Fa	LSP
19	My	Lo	Ne	LSP
20	My	Lo	Mo	VLSP
21	My	Lo	Fa	VLSP
22	My	Mdm	Ne	MSP
23	My	Mdm	Mo	VLSP
24	My	Mdm	Fa	VLSP
25	My	Hi	Ne	VHSP
26	My	Hi	Mo	LSP
27	My	Hi	Fa	LSP

**Table 3:** FRB2.

No.	TR	DE	RE	NSA	ASD	No.	TR	DE	RE	NSA	ASD
1	Sh	Ne	Lo	Fw	VLSP	41	Mi	Mo	Mdm	Me	VHSP
2	Sh	Ne	Lo	Me	HSP	42	Mi	Mo	Mdm	My	HSP
3	Sh	Ne	Lo	My	VLSP	43	Mi	Mo	Hi	Fw	VHSP
4	Sh	Ne	Mdm	Fw	MSP	44	Mi	Mo	Hi	Me	VHSP
5	Sh	Ne	Mdm	Me	VHSP	45	Mi	Mo	Hi	My	VHSP
6	Sh	Ne	Mdm	My	MSP	46	Mi	Fa	Lo	Fw	VLSP
7	Sh	Ne	Hi	Fw	HSP	47	Mi	Fa	Lo	Me	MSP
8	Sh	Ne	Hi	Me	VHSP	48	Mi	Fa	Lo	My	VLSP
9	Sh	Ne	Hi	My	VHSP	49	Mi	Fa	Mdm	Fw	LSP
10	Sh	Mo	Lo	Fw	VLSP	50	Mi	Fa	Mdm	Me	VHSP
11	Sh	Mo	Lo	Me	LSP	51	Mi	Fa	Mdm	My	LSP
12	Sh	Mo	Lo	My	VLSP	52	Mi	Fa	Hi	Fw	MSP
13	Sh	Mo	Mdm	Fw	VLSP	53	Mi	Fa	Hi	Me	VHSP
14	Sh	Mo	Mdm	Me	HSP	54	Mi	Fa	Hi	My	HSP
15	Sh	Mo	Mdm	My	LSP	55	Ln	Ne	Lo	Fw	VLSP
16	Sh	Mo	Hi	Fw	MSP	56	Ln	Ne	Lo	Me	HSP
17	Sh	Mo	Hi	Me	VHSP	57	Ln	Ne	Lo	My	LSP
18	Sh	Mo	Hi	My	MSP	58	Ln	Ne	Mdm	Fw	MSP
19	Sh	Fa	Lo	Fw	VLSP	59	Ln	Ne	Mdm	Me	VHSP
20	Sh	Fa	Lo	Me	VLSP	60	Ln	Ne	Mdm	My	HSP
21	Sh	Fa	Lo	My	VLSP	61	Ln	Ne	Hi	Fw	VHSP
22	Sh	Fa	Mdm	Fw	VLSP	62	Ln	Ne	Hi	Me	VHSP
23	Sh	Fa	Mdm	Me	MSP	63	Ln	Ne	Hi	My	VHSP
24	Sh	Fa	Mdm	My	VLSP	64	Ln	Mo	Lo	Fw	VLSP
25	Sh	Fa	Hi	Fw	VLSP	65	Ln	Mo	Lo	Me	MSP
26	Sh	Fa	Hi	Me	HSP	66	Ln	Mo	Lo	My	VLSP
27	Sh	Fa	Hi	My	LSP	67	Ln	Mo	Mdm	Fw	LSP
28	Mi	Ne	Lo	Fw	MSP	68	Ln	Mo	Mdm	Me	VHSP
29	Mi	Ne	Lo	Me	VHSP	69	Ln	Mo	Mdm	My	LSP
30	Mi	Ne	Lo	My	HSP	70	Ln	Mo	Hi	Fw	MSP
31	Mi	Ne	Mdm	Fw	VHSP	71	Ln	Mo	Hi	Me	VHSP
32	Mi	Ne	Mdm	Me	VHSP	72	Ln	Mo	Hi	My	HSP
33	Mi	Ne	Mdm	My	VHSP	73	Ln	Fa	Lo	Fw	VLSP
34	Mi	Ne	Hi	Fw	VHSP	74	Ln	Fa	Lo	Me	VLSP
35	Mi	Ne	Hi	Me	VHSP	75	Ln	Fa	Lo	My	VLSP
36	Mi	Ne	Hi	My	VHSP	76	Ln	Fa	Mdm	Fw	VLSP
37	Mi	Mo	Lo	Fw	LSP	77	Ln	Fa	Mdm	Me	MSP
38	Mi	Mo	Lo	Me	VHSP	78	Ln	Fa	Mdm	My	VLSP
39	Mi	Mo	Lo	My	LSP	79	Ln	Fa	Hi	Fw	LSP
40	Mi	Mo	Mdm	Fw	HSP	80	Ln	Fa	Hi	Me	HSP
						81	Ln	Fa	Hi	My	LSP

**Table 4:** Experimental Parameters

Experimental Parameters	
Operating System	MacOS, Ubuntu 18.04
Experiment Area	Indoor 2 × 2 (m^2^)
Number of Actors	3 [Arduino Uno]
Number of Sensors	3 [TR, Battery, Distance]
Number of Events	25
IDE	Arduino IDE v. 1.8.8, Processing v. 3.5.3
